# Enhancing sustainability in soldering: effects of recycled welding slag and rice husk ash on microstructural, thermal, and mechanical properties of Sn–Cu composite solders

**DOI:** 10.1038/s41598-025-27369-z

**Published:** 2025-11-28

**Authors:** H. N. Soliman, H. Gassour, Mohamed Morsy, Amir Elzwawy, M. Amin, M. Ali

**Affiliations:** 1 Department of Physics, Faculty of Education, Ain Shams University, Heliopolis, Roxy, PO Box 5101, Cairo, 11771 Egypt; 2 Department of Mechanical Engineering, Higher Technological Institute (HTI), 10th of Ramadan City, 44629 Egypt; 3 Department of Mech. Eng American University in Cairo, Cairo, Egypt; 4 Building Physics and Environment Institute, Housing & Building National Research Center (HBRC), Dokki, Giza, 12311 Egypt; 5 Nanotechnology Research Center (NTRC), The British University in Egypt (BUE), El-Sherouk City, Cairo 11837 Egypt; 6 Refractories, Ceramics and Building Materials Department, Advanced Materials Technology and Mineral Resources Research Institute, National Research Center (NRC), 33 El Bohouth St., Dokki, Giza, 12622 Egypt; 7 Physics Department, Faculty of Science, Galala University, New Galala City, Suez, 43511 Egypt

**Keywords:** Solder alloys, Recycled welding slag, Rice husk ash, Sustainable development, Mechanical properties, Engineering, Materials science

## Abstract

This study investigated the microstructural, thermal and mechanical properties of a series of plain and composite lead-free solder systems (Sn–0.7Cu − xWS; Sn–0.7Cu − xRHA; x = 0, 6, and 12 wt%), as well as sustainable reinforcing solutions. The microstructural characteristics of the present alloys were investigated using scanning electron microscopy (SEM), energy dispersive X-ray spectrometry (EDS), electron probe microanalysis (EPMA), and X-ray diffraction (XRD). Moreover, thermal properties of the present composites were investigated utilizing differential scanning calorimetry (DSC). Images obtained from the SEM demonstrate that welding slag (WS) and rice husk ash (RHA) nanoparticles are uniformly distributed within the composite material. The findings showed that the incorporation of WS and RHA nanoparticles markedly altered the microstructure of these alloys and improved their thermal and mechanical properties. It was observed that the mechanical properties of the Sn-0.7Cu-xRHA composites were superior to those of the Sn-0.7Cu-xWS composites. Additionally, compared to other composites, the Sn-0.7Cu-6RHA composites exhibited significantly higher levels of tensile strength and hardness, while the Sn-0.7Cu-12RHA composites revealed superior thermal characteristics. It also focused on the environmental benefits and potential challenges of using recycled agricultural waste and welding slag in Sn-0.7Cu solders from a life cycle assessment perspective.

**Supplementary Information:**

The online version contains supplementary material available at 10.1038/s41598-025-27369-z.

## Introduction

Recycling and optimizing materials have become crucial in modern engineering due to sustainability concerns and resources shortage. In the case of Tin-Copper alloys (Sn–Cu), which are widely used in many applications, such as electronic components, soldering, and structural materials, improving their mechanical properties as well as thermal characteristics through recycled reinforcements is an area of significant research interest^1,2^.

The mechanical performance of Sn–Cu alloys depends on factors such as composition, microstructure, and percentage of reinforcement’s addition^3^. While primary reinforcements are often used to improve properties like strength, hardness, and wear resistance, recycling and reusing reinforcements present a cost-effective and environmentally friendly alternative^2^. However, the challenge lies in maintaining or even improving mechanical characteristics while incorporating sustainable solutions.

Tin alloys (Sn) have gained significant attention in industrial applications due to their excellent mechanical properties, corrosion resistance, and low melting point^4,5^. These alloys are commonly used in soldering, bearings, and coatings. However, improving their casting properties, such as fluidity, porosity reduction, and mechanical strength, remains a challenge^6,7^. A promising approach to enhance these properties is the incorporation of eco-friendly reinforcements derived from recycled agricultural waste and industrial by-products such as welding slag^7,8^.

Recycled agricultural waste, including rice husk ash (RHA), coconut shell ash (CSA), and sugarcane bagasse ash (SCBA), contain high amounts of silica (SiO_2_), alumina (Al_2_O_2_), and other oxides that can enhance the mechanical and tribological properties of Sn alloys^9,10^. Similarly, welding slag (WS), a by-product of welding operations, is rich in metallic oxides and can act as a reinforcing agent to improve the structural integrity of Sn alloys when used in casting^1^. Researchers have started exploring the potential benefits of integrating these waste materials into Sn alloys to enhance their casting properties, mechanical performance, and environmental sustainability.

In 2020, Sahin and Murphy investigated the potential use of silica-based Agro-reinforcements. They discovered that RHA that contains up to 90% silica can refine the grain structure and enhance material hardness when incorporated into metal matrices. They also concluded that agricultural waste-derived reinforcements have been widely used to reinforce aluminum and copper-based composites, demonstrating improvements in hardness, wear resistance, and thermal stability^9^. Similarly, CSA and SCBA, investigated by Abubakar et al. in 2021, are known to provide improved wear resistance due to their carbon and silica-rich composition^11^. These reinforcements, when used in Sn-based alloys, boosted the grain refinement process by enhancing microstructure uniformity and reducing casting defects^12^. It also improved hardness and wear resistance due to the presence of ceramic oxides^13^. Eco-friendly material utilization: Reducing industrial waste and promoting sustainability^14^.

Welding slag is primarily composed of Fe_2_O_2_, SiO_2_, MnO, and other oxides^14,15^. It has been used as a reinforcement to composite materials due to its ability to improve mechanical properties and wear resistance. Previous studies on WS-reinforced composites have shown much progress. In 2023, G. Venkatramana and B. Vidivelli studied the effects of using slag to reinforce concrete in building blocks. They reported an improvement in modulus of elasticity by 15% when using 30% steel slag reinforcements.

In 2018, Ramesh et al. investigated the sustainable use of (WS) to reinforce metallic alloys. They reported increased hardness and tensile strength in metallic matrices. Later, in 2021, Chandramohan D. and others studied metallic slag composites and introduced it as a novel route for cost-effective and high-performance reinforcements. Improved thermal stability and wear resistance were identified, making it suitable for casting and high temperature applications^16^. They outlined a remarkable reduction in manufacturing costs by effectively using such industrial waste^17^.

By incorporating (WS) into Sn alloys, casting properties can be enhanced by reducing shrinkage and porosity, leading to better mechanical integrity, as stated by Hasan T. and others in 2024^18^. They also concluded that there was an adequate increase in thermal resistance, which is crucial for soldering applications and for improved wetting behavior, allowing for improved structure bonding.

Although the addition of recycled agricultural waste and welding slag to Sn alloys presents numerous advantages, many challenges must be carefully addressed. The environmental/economic impact summarizes the long story of sustainable industry and should be scientifically considered^19^. The uniformity of reinforcement dispersion within the metal matrix has always gained researchers’ attention as it forms an important challenge that requires advanced stirring and proper mixing techniques^20,21^. Moreover, the compatibility challenge between Sn and ceramic reinforcements must be optimized to prevent brittle phases.

From the past mentioned and unmentioned literature, many investigation fields have yet to be fulfilled. These unfilled research gaps may be summarized by the following questions that formed our research objectives:

- How do recycled agricultural waste and welding slags interact with the Sn–Cu matrix at the microstructural level, and how do these reinforcements influence the spreading behavior, wettability, and formation of intermetallic compounds (IMC) in Sn-Cu soldering applications?
- How are the mechanical/thermal properties affected by Agro-waste and recycled slag compared to conventional reinforcements?
- What challenges arise when integrating such reinforced Sn–Cu solders into conventional soldering applications?
- What are the environmental benefits and potential trade-offs of using recycled agricultural waste and (WS) in Sn–Cu solders from a life-cycle assessment perspective?

This study focuses on the optimization of recycled reinforcements to improve the mechanical properties of Sn–0.7Cu alloys. By introducing a novel casting technique, reinforcement weights, and types, then analyzing their effects on tensile strength, hardness, and microstructure. This research aims to develop a systematic approach to achieve high performance recycled Sn–Cu composites. For that reason, advanced characterization techniques such as scanning electron microscopy (SEM), XRD, and mechanical testing will be employed to evaluate the structural integrity and mechanical behavior of the optimized materials.

## Materials and methods

The purpose of the research is to optimize the potential use of recycled resources to enhance the physical and mechanical properties of the Sn–0.7Cu alloy. For this purpose, casting was used to prepare the test samples needed to evaluate the targeted enhancement. The experimental research work was divided into two main sections, namely samples preparing and samples testing.

### Samples preparation

#### Materials

One kilogram of raw tin (Sn) was purchased from Sigma-Aldrich with purity of 99% and copper wire (purity 95%) was purchased from local market (El-sewedy company). The two reinforcements used were collected from industrial waste (Welding Slag) and agricultural waste (Rice Husk Ash) and will be referred to as WS and RHA respectively.

#### Reinforcement preparation

Tin was cut into small pieces, then copper wire was cut into small cuts and was weighed using sensitive balance to prepare the predetermined mixing ratio of (99.3 Sn: 0.7Cu) by weight, which is equivalent to (148.95 g Sn: 1.05 g Cu). The base alloy was cast, and reference samples were prepared according to the required physical and mechanical testing standards and procedures (ASTM E8/E8M, D-8328, F-1372, E-384), as mentioned later. The RHA was collected and burned at 600 °C and then refined and converted to fine powder. The recycled WS consists mainly of metal oxides (e.g., iron and silica, as characterized later by XRD and TEM analysis) produced as a byproduct of welding processes, was collected from industrial wastes, washed properly using warm water and detergent to remove any impurities, and then dried. Then it was milled to fine powder. Subsequently, both reinforcements were identified using an X-ray diffraction (XRD) test before reinforcing the base alloy.

#### Samples preparation

The reinforcements, RHA and WS, were weighed and assigned as nominal percentages for research samples’ reinforcement. The first group, reinforced by agriculture waste RHA, consisted of three samples, namely: Sn + 0.7Cu base alloy, base alloy + 6% RHA, and base alloy + 12 wt% RHA by nominal weights. The second group, reinforced by (WS), consisted of the same mixing nominal ratios as indicated in Table 1.

**Table 1:** Chemical composition of Sn–Cu-x (WS, RHA) composite solders in nominal wt%.

Alloy	Cu (wt%)	WS (wt%)	RHA (wt%)	Sn (wt%)
**S**_**0**_	0.7	–	–	Bal.
**S**_**1**_	0.7	6	–	Bal.
**S**_**2**_	0.7	12	–	Bal.
**S**_**3**_	0.7	–	6	Bal.
**S**_**4**_	0.7	–	12	Bal.

### Casting process

The sample casting was performed according to ASTM -E88 for sampling non-ferrous metals and alloys. Tin was first melted in the furnace due to its low melting point of 231.9 °C, then copper was gradually added with continuous stirring to ensure uniform particle distribution.

The temperature is maintained at 1200 °C, above the copper’s melting point of copper of 1085 °C to ensure proper melting^22^. Pre-allocated reinforcements mentioned in Table 1, were added for each sampling mixture at an approximate rate of 1 g/min to ensure proper dispersion. The molten mixture was then poured into a pre-prepared mold and left to free convection cooling. Stirring was performed carefully to ensure that RHA and WS particles are fully integrated into the molten alloy.

All samples were ground and polished to obtain the required surface finish. The grinding was carried out in three stages: Rough grinding, using a coarse-grit abrasive wheel or belt (e.g. 120–240 grit) to remove large imperfections and surface oxides, then intermediate grinding, a medium-grit abrasive (e.g. 400–600 grit), for further refinement of the surface to ensure the surface is uniformly smooth before moving to the final stage of fine grinding, a fine-grit abrasive (e.g. 800–1200 grit), to create a uniform and smoothly finish surface that is ready for polishing process. The polishing process was carried out to enhance surface roughness and corrosion resistance. A suitable polishing compound, diamond paste 1- micron, was used and the process was carried out in three stages to produce fine and smooth surface finish. After the polishing process, chemical etching was performed by immersing the samples in the following etchant (HNO_3_ 2% wt. + HCL 3% wt. + C_2_H_5_OH 95% wt.) for 15 s. in preparation for microstructure inspection.

### Testing samples

For proper evaluation of research purposes, several tests were performed considering the scientific standards that allowed us to judge the claimed enhancements. SEM, TEM, and optical photography were used to ensure that the casting process was intact, and the produced samples microstructure is improved, ensuring even distribution of all additives within the produced alloy. Regarding mechanical characteristic enhancements, the Tensile and Hardness tests were enough to justify the positive contribution gained by the sustainable utilization of agriculture and recycled waste.

#### Optical microscope

High-power photomicrographic equipment, with the model MF-AKS with two eyepieces, equipped with a stereo microscope is used to create a 3-D effect. A camera is utilized to capture images with 20×. Alternatives to optical microscopy which do not use visible light are scanning electron microscopy and transmission electron microscopy which will also be used to achieve much greater magnification.

#### Transmission electron microscopy (TEM)

The used TEM device model is JEM-100CX, which was primarily used to characterize the recycled powders prior to dispersion in the reinforcing process. It helped to reveal the atomic arrangement and helped identify crystalline defects and voids/dislocations. It also helped identify the boundaries of granules or additives. The test was performed according to ASTM D-8328 standards to ensure proper conclusions.

#### XRD (XRD)

The International Center for Diffraction Data (ICDD) database of X-ray diffraction patterns enables the phase identification of a large variety of crystalline samples. XRD data was collected using a Malvern Panalytical Empyrean-3 diffractometer with the Cu K radiation source Cu Kα (λ = 1.5406 Å). For each prepared sample, data were collected from 2θ = 5–90° at a scan rate of 5/min. The peak intensities were used to calculate FWHM and relative abundance of each phase in the sample.

#### Scanning electron microscope (SEM)

SEM examination and EDX analysis were performed using Quanta FEG 250 scanning electron microscope (SEM) equipped with an EDX unit. It should be noted that samples microstructural analysis of the samples performed according to the ASTM-F1372 standard boosted the reliability of the results as it formed a common ground for comparison and evaluation.

#### Tensile test

The tensile test is performed to measure strength and ductility performance utilizing universal electronic tensile testing equipment (model LFM-20KN, BENCH TOP). A standardized sample, 80 mm in length and 7 mm in diameter, was prepared to ensure consistent results. The specimen is then mounted on the tensile testing machine and a proper uniaxial tensile constant strain rate of $\:1.7\times\:{10}^{-4}{\:\text{s}}^{-1}\:$ is selected and applied to the fracture. This strain rate was chosen in accordance with standard testing protocols for soft metallic composites (e.g. ASTM E8/E8M), ensuring consistency of results. To ensure reproducibility and improve confidence level, three specimens per casting were tested and the average values of tensile properties were recorded. The results were then processed using Origin software to estimate some characteristics of mechanical performance.

#### Micro-hardness test

Microhardness testing is used to measure the improvements in hardness of reinforced Sn–0.7Cu alloy on a microscopic scale. Measurements were made according to ASTM E384 using a Digital Micro Vickers Hardness Tester (Model: Bht 1000) loaded with 1KgF and a dwelling time of 13 s. This test was characterized by the small indentation suitable for our test samples, precise load control, and the microscopic indentation evaluation. To ensure accuracy, five independent measurements per sample were taken, and the average value was calculated and reported.

## Results and discussion

### Characteristics of welding slag (WS) and rice husk ash (RHA)

**Fig. 1:**
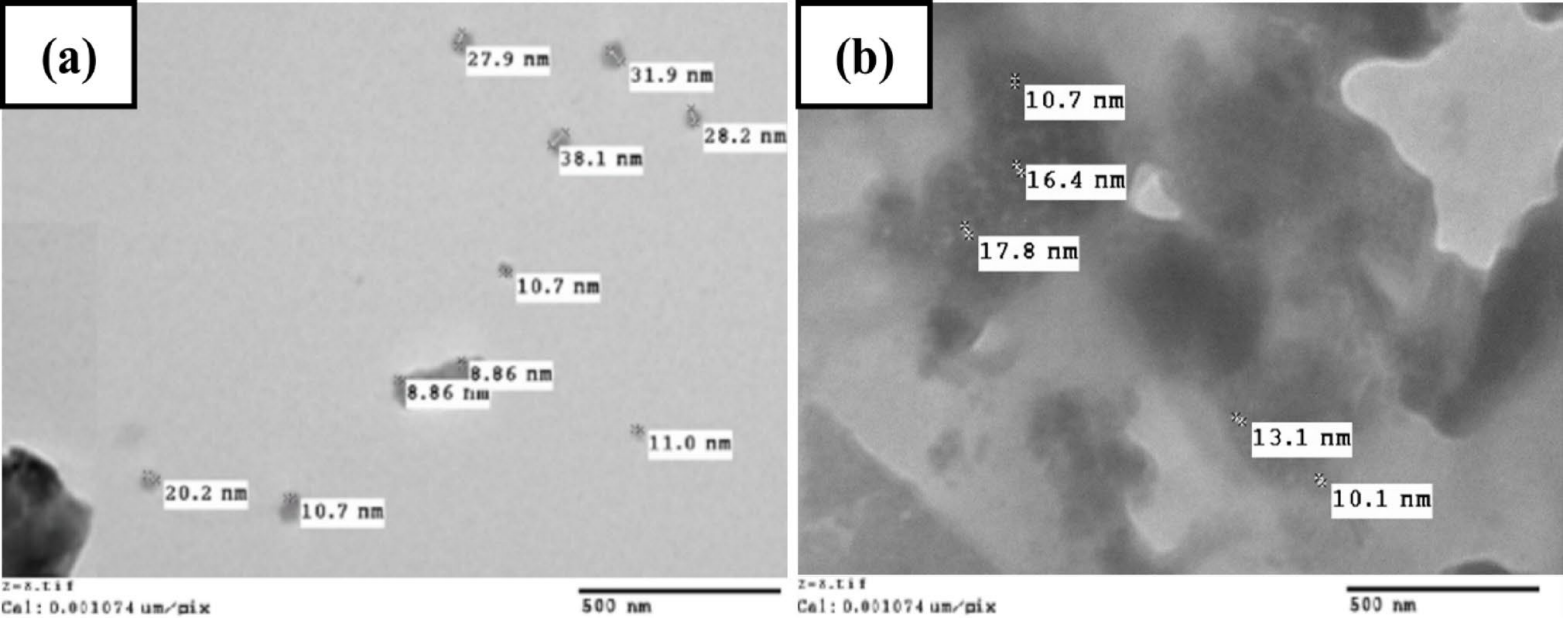
TEM images of reinforcements: (**a**) Welding slag and (**b**) Rice husk ash.

The size, shape and distribution play a vital role in determining the mechanical properties of the tin-metal matrix composites. Figure 1 shows the results of the particle size analysis for the powder of WS and RHA used in this study.

 TEM micrographs presented in Fig. 1a,b confirm the presence of the WS and RHA nanoparticles within the Sn–0.7Cu solder alloy. The observed particle sizes range between 8 and 36 nm, which is consistent with the nanoscale dimensions expected for these reinforcements. Specifically, Fig. 1a reveals relatively discrete and well-defined nanoparticles, attributable to WS, while Fig. 1b shows slightly agglomerated, but clearly distinguishable particles, characteristic of RHA-derived silica phases. Furthermore, complementary FE-SEM analyzes and EDS mapping of the S1, S2 and S3 composites (Fig. 6b–d) substantiate that these reinforcement particles are uniformly distributed throughout the solder matrix, without evidence of gaps, clustering, or discontinuities. This homogeneous distribution indicates successful dispersion and strong interfacial bonding between the Sn–0.7Cu matrix and the nanoparticles, which is believed to be essential for the anticipated improvements in the microstructural stability and mechanical properties of the solder.

**Fig. 2:**
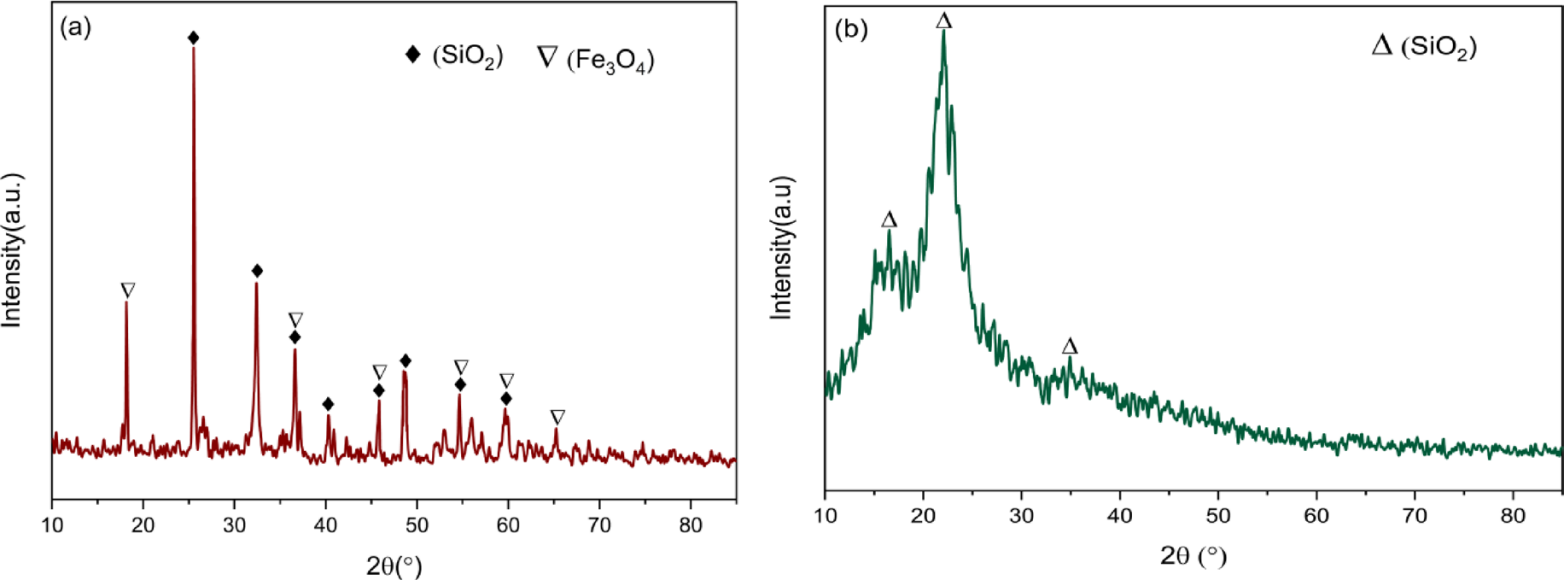
XRD patterns of reinforcements: (**a**) Welding slag and (**b**) Rice husk ash.

The average particle size of the WS powder is 20.77 nm, with the lower and higher values of 7.52 and 35.6 nm, respectively. Furthermore, the average particle size of the RHA powder is 13.62 nm, with lower and higher values of 10.1 and 17.8 nm, respectively. The crystal structures of WS and RHA used in this research have been validated later by (XRD) as shown in Fig. 2.

Initially, the XRD diffractogram of the welding slag (Fig. 2a) represents the inclusion of Fe and Si as the main components of the structure. The characteristic peaks are located at 24.1° (101), 31.1° (111), 36.7° (102), 40.1° (200), 45.6° (211), 48.7° (113), 54.7° (212) and 59.5° (004) are designated to the tetragonal SiO_2_ with the card no. 01-080-3754 revealing the angles of α, β, γ = 90°, while a = b = 4.5, c = 6.1.

The main peak for SiO_2_ is located near 25°. Other phases in the welding slag are attributed to cubic Fe_3_O_4_ where the main peaks are delivered as 18.1° (111), 36.5° (311), 45.7° (400), 54.4° (422), 59.5° as per the XRD card number. 01-084-6694. Other Fe phases such as Fe_2_O_3_ might be distinguished in welding slag with the main peak located near 35° and overlapping with other Fe peaks.

The XRD diffractogram of the rice husk ash (Fig. 2b) is mainly composed of amorphous silicon dioxide. Inherently, there is one broad hump mounting from ~ 20–25° with negligible extra peaks. This broad hump peaked around 22.1°, which is attributed to the (120) diffraction plane for amorphous SiO_2_. As stated for amorphous silica with relevant JCPDS card number of 29-0085.

### Thermal behavior analysis

**Fig. 3:**
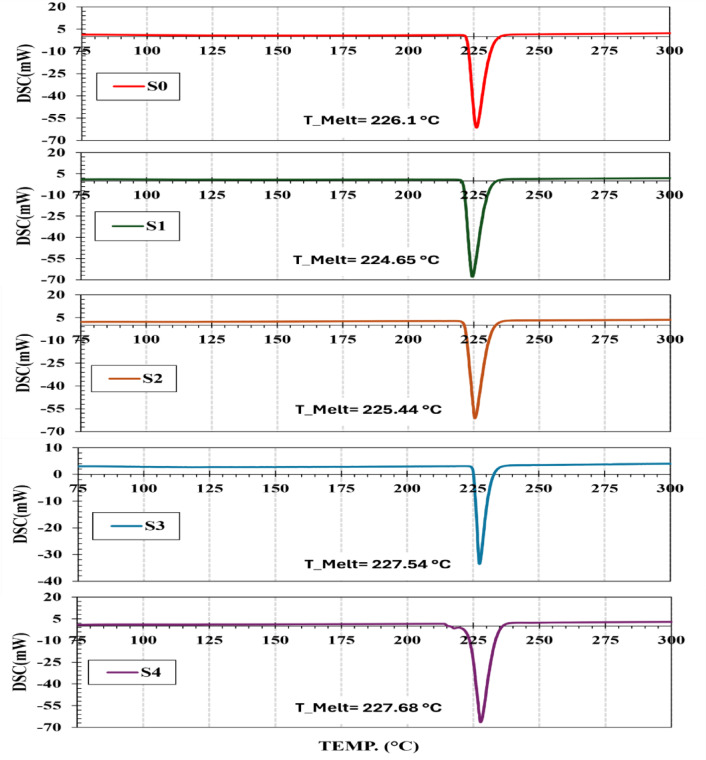
DSC profiles of: (**a**) S0, (**b**) S1, (**c**) S2, (**d**) S3, and (**e**) S4 composites.

The melting temperature of a solder interconnect is a critical parameter to ensure compatibility with the operating temperature of electronic devices and the thermal limits of integrated IC components. It is also imperative to assess the influence of sustainable reinforcements such as Agro-waste (RHA) and (WS), which are increasingly considered in green manufacturing. Figure 3 presents the differential scanning calorimetry (DSC) profiles of the plain and composite solder samples, while the associated thermal parameters, melting temperature (Tm), solidus temperature (Ts), liquidus temperature (TL), pasty range (ΔT), and enthalpy of fusion (ΔH) are summarized in Table 2.

**Table 2:** Comparison of melting temperature (Tm), solidus temperature (Ts), Liquidus temperature (TL), pasty range (ΔT), and fusion enthalpy of fusion (ΔH) for plain and composite solder systems.

Alloy	T_m_ (°C)	T_s_ (°C)	T_L_ (°C)	ΔT = T_L_-T_s_ (°C)	ΔH (Mw·°C)	Normalized ΔH factor
**S**_**0**_	226.10	216.29	238.41	22.12	561.65	1.00
**S**_**1**_	224.65	214.41	238.55	24.14	521.45	0.93
**S**_**2**_	225.44	214.75	238.22	23.47	349.75	0.62
**S**_**3**_	227.54	217.20	238.93	21.73	597.97	1.07
**S**_**4**_	227.68	214.37	241.41	27.04	605.19	1.08

Compared to the plain solder alloy (S_0_), which has a T_m_ of 226.10 °C, both S_1_ (224.65 °C) and S_2_ (225.44 °C) show slightly reduced melting points. On the contrary, S_3_ (227.54 °C) and S_4_ (227.68 ° C) show slightly higher melting temperatures. This shift can be attributed to the type and concentration of reinforcements used.

The solidus temperatures followed a similar trend. The S1 and S_2_ compounds exhibited values of 214.41 °C and 214.75 °C, respectively, which are slightly lower than the plain solder S_0_ (216.29 °C). However, S_3_ showed a higher T_s_ of 217.20 °C, while S_4_ displayed a slightly lower value of 214.37 ° C. Regarding the liquidus temperature, S_1_ (238.55 °C) and S_2_ (238.22 °C) are comparable to S_0_ (238.41 °C), while S_3_ and S_4_ registered higher T_L_ values (238.93 °C and 241.41 °C, respectively).

The pasty range (ΔT = T_L_ – T_s_) is crucial for solderability and solidification dynamics. S_1_ and S_2_ demonstrated moderately widened pasty ranges of 24.14 °C and 23.47 °C compared to 22.12 °C for plain solder. In particular, the S_4_ compound exhibited the broadest pasty range (27.04 °C), indicating prolonged semisolid behavior during cooling, which may increase the probability of porosity or hot tearing due to shrinkage and differential thermal contraction^4^.

In terms of enthalpy of fusion (ΔH), quantitative inspection of the area of the endothermic peaks of DSC indicates lower ΔH for S_1_ and S_2_ as indicated by the normalization factor (0.93, 0.62, respectively), suggesting lower energy requirements for phase change. On the contrary, the more intense peaks for S_3_ and S_4_ signify higher ΔH values as indicated by the normalization factor (1.07, 1.08 respectively), pointing to improved microstructural homogeneity and thermal stability.

This trend may be related to the better dispersion and wetting behavior of the reinforcing particles, especially at higher addition ratios. Such results support the correlation between reinforcement amounts and distribution integrity with thermal behavior. These results are consistent with previous findings that observed shifts in thermal transitions after reinforcing Sn-based solder matrices with nano oxides such as TiO_2_^3^. The lowest T_m_ recorded in the S_1_ system (12 nominal wt% WS) also implies its suitability in low-temperature applications and confirms its viability for sustainable materials design in electronics packaging.

### X-ray analysis

XRD analyzes were conducted to explore the preliminary characteristics of the intended structures and the impact of the incorporation of welding slag (WS) and rice husk ash (RHA) within the Sn–0.7Cu matrix. The analysis shows how RHA and WS interact with the Sn–Cu matrix at the atomic level, and how these reinforcements influence the spreading behavior and formation of intermetallic compounds (IMCs) in Sn–0.7Cu soldering alloys.

**Fig. 4:**
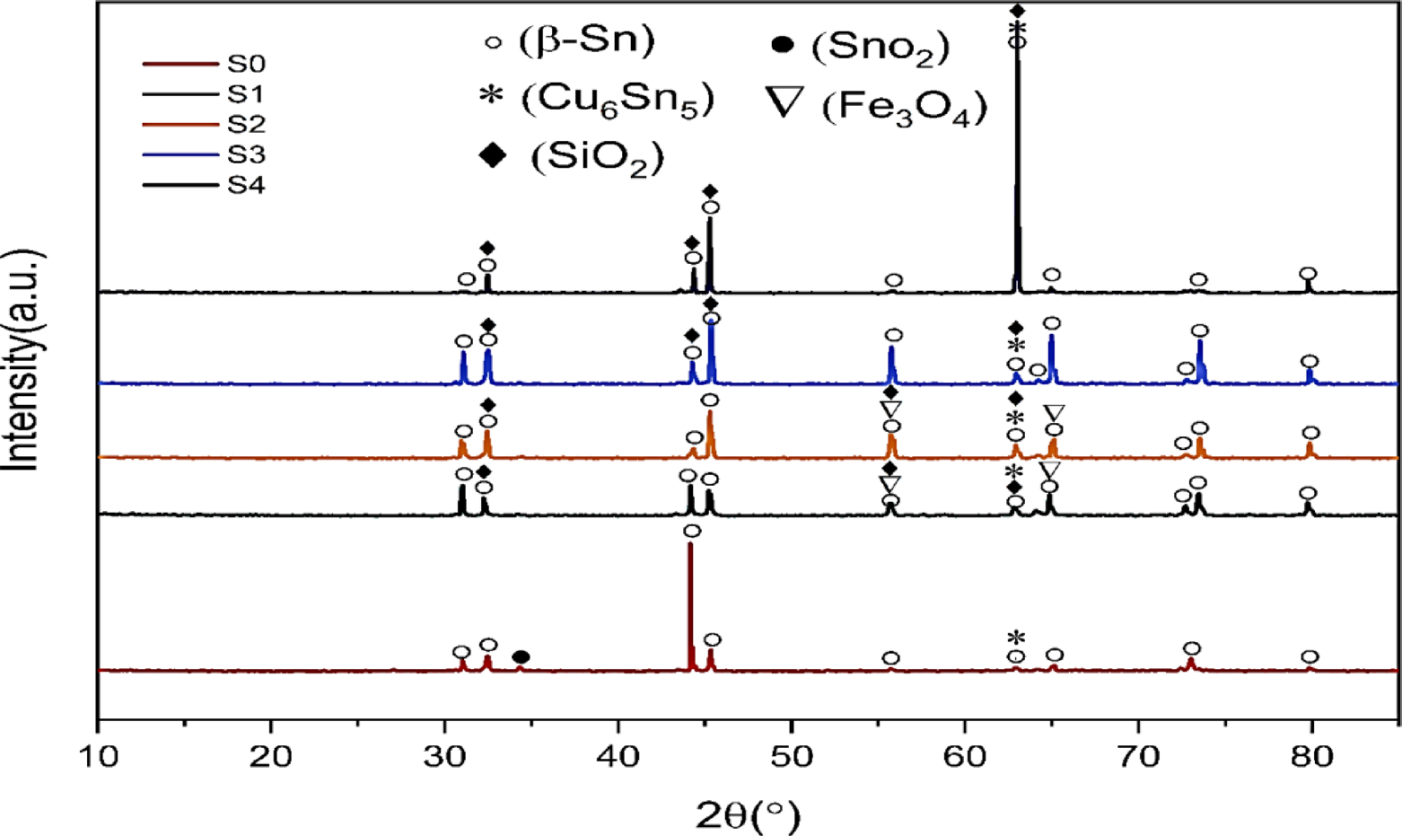
XRD patterns for (**a**) S0, (**b**) S1, (**c**) S2, (**d**) S3, and (**e**) S4 composites.

The samples are defined as WS, RHA, S_0_, S_1_, S_2_, S3 and S_4_. The diffraction patterns for the synthesized structures are depicted in Fig. 4. The diffraction peaks reflect the purity of the prepared alloy where the most intense peaks reside at 30.9° (200), 32.6° (101), 44.3° (220), 45.4° (211), 55.2° (301), 62.9° (112), 65.2° (321), 73.1° (420), and 79.8° (312) with β-Sn as the main phase of the Sn–0.7Cu alloy. These peaks and the corresponding diffraction planes are mentioned in card no. 01-085-5861 having a negligible shift (< 1°). A minor peak for SnO_2_ exists at 34.4 ° (110) as recorded on card no. 01-071-4746. The tiny amount of Cu in the Sn matrix produces a minor overlapping peak for the Cu_6_ Sn_5_ phase at 62.9° (112) as represented in Fig. 4a. The dominance of the tetragonal phase is affirmed for the Sn-Cu alloy having lattice parameters of a = b = 5.8 Å, c = 3.2 Å, while α = β = γ = 90° as determined by the card no. 01-085-5861. All detected diffraction peaks were intense and sharp, which revealed that the synthesized alloy is abundantly crystallized in nature^23^.

Figure 4b,c represents the addition of effect of the welding slag to the plain Sn–-0.7Cu alloy. On the one hand, the original peaks for the plain Sn–0.7Cu alloy are sharpened upon the inclusion of 6%WS without any remarkable shift or upsurge of impurities and slag peaks. This might be regarded to the physical nature of the slag which converges with the initial alloy matrix and resides homogeneously in the matrix of the plain Sn–0.7Cu alloy. It is worth mentioning that the main peak of the Sn–0.7Cu alloy inhibiting at 44.3° was reduced in intensity. An additional increase in WS to 12 wt% increased the peak intensities of the Fe_3_O_4_, SiO_2_ and Cu_6_Sn_5_ phases (Fig. 4b,c). Due to the well-ordered and distributed nature of the WS particles within the main matrix composition, the Sn–0.7Cu-WS alloy structure revealed expected better order and crystallinity.

Incorporation of RHA ash into the Sn–0.7Cu matrix follows the same trend as the incorporation of the WS into the matrix. The conjugation between the obtained RHA and the prepared Sn–0.7Cu alloy with two different nominal concentrations of RHA (6, 12 wt%) is shown in Fig. 4d,e. At first glance, the pattern retained the parent crystal structure, and an escalation of the low-intensity peaks for the Sn–Cu alloy is noticed.

The resident peaks located around 31.0°, 32.4°, 45.3°, 55.8°, 62.9°, 64.9°, 73.6°, and 79.8° were intensified, while the inhibiting main peak of the Sn–0.7Cu at 44.3° was decreased as remarked earlier in the welding slag. An additional increase in RHA to 12 wt% augmented the peak intensities of the SiO_2_ and Cu_6_Sn_5_ phases (Fig. 4d,e).

This approach suggests the successful incorporation of RHA into the Sn-0.7Cu alloy, because of the substitution of Sn atoms by C atoms to modify the Cu_6_ Sn_5_ phase. Growth of the carbonic structure suppresses the order of the alloy and opposes the highest peak of the Sn, offering the successful incorporation and replacement of the Sn. Truly, the degree of solidification and nucleation has a straight influence on the peak intensity^5^. The decline of the main Sn peak is reported in former research^5,24^. Inclusion of the RHA in plain Sn–0.7Cu solder maintaining the location of the mother peaks without the noticeable shift. This suggests the homogeneity of the prepared Sn–0.7Cu alloy and the successful incorporation of the mixing through the frequent melting and casting process.

It is worth noting that the addition of non-reactive WS and RHA nanoparticles into the Sn–0.7Cu matrix increases the intensity of Cu_6_ Sn_5_ planes, while the intensities of β-Sn planes decrease. This implies that adding WS and RHA nanoparticles to the current Sn–0.7Cu solder has a synergetic influence on the growth of the Cu_6_Sn_5_ phase. This can be explained as follows: The agglomeration of the WS and RHA nanoparticles inside the alloy matrix increased the interaction between Cu and Sn atoms, and accordingly more Cu_6_Sn_5_ phase was found. For further insights into a few parameters for the formed structures, the crystallite size and the dislocation densities are validated mathematically. The crystallite size (D) is determined following the Sherrer equation as^25,26^:

1 $$\:{D}\left({n}{m}\right)=\left(\frac{{k}{*}{\lambda\:}}{{\beta\:}{*}{C}{o}{s}{\theta\:}}\right)$$

The k, λ, β, and θ are denoted as the shape factor, the incident radiation wavelength, the FWHM, and the diffraction angle, respectively. Taking these values in radians, the crystallite size can be determined. The FWHM is considered depending on the most intense peak of the structure.

The dislocations density (δ) can be extracted relying on the crystallite size as^26,27^:

2 $$\:{\delta\:}={N}{*}{{D}}^{-2},\:{N}=1$$

The extracted values are tabulated in Table 3. This table demonstrates that the crystallite sizes in the S_1_, S_2_, S_3_, and S_4_ composites are smaller than those of the plain solder S_0_, although the dislocation densities of the S_1_, S_2_, S3 and S_4_ composites exceed those of the plain solder S_0_.

**Table 3:** The parameters obtained for the acquired structures.

Alloy	FWHM (°)	2θ (°)	D (nm)	δ (nm^− 2^)
S_0_	0.03	44.07	298.50	1.12 × 10^− 5^
S_1_	0.22	30.9	39.15	6.52 × 10^− 4^
S_2_	0.23	45.3	39.11	6.54 × 10^− 4^
S_3_	0.19	45.2	47.32	4.47 × 10^− 4^
S_4_	0.05	62.9	194.62	2.64 × 10^− 5^

### Microstructural characterization

#### Optical analysis

**Fig. 5:**
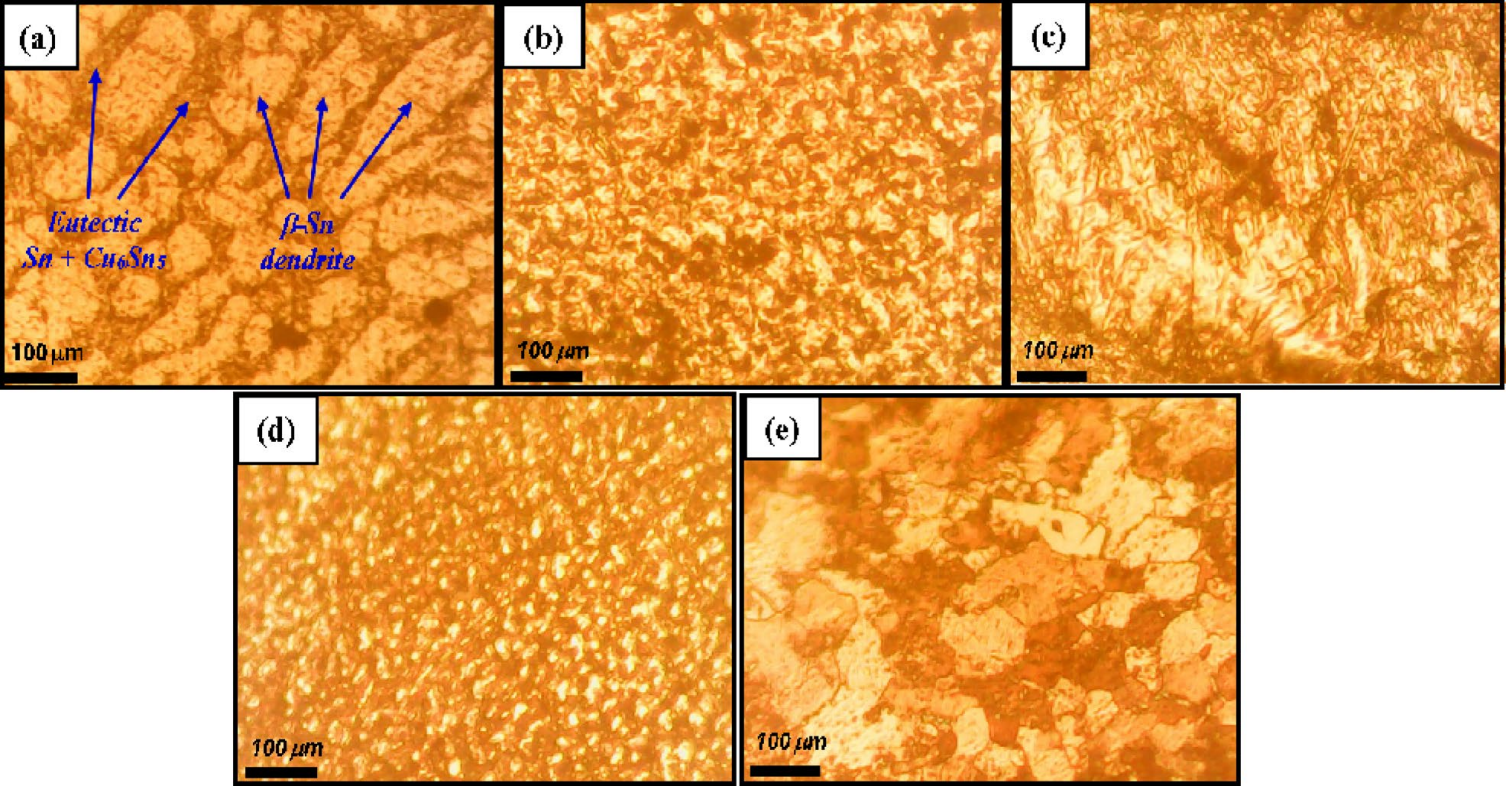
Optical micrographs of (**a**) S_0_, (**b**) S_1_, (**c**) S_2_, (**d**) S_3_, and (**e**) S_4_ composites.

Figure 5 shows optical micrographs of the composites examined. While the chosen reinforcements, RHA and WS, are ceramic materials with high thermal stability, poor wettability, and limited reactivity at lower temperatures, casting temperatures of 1200 °C are used to promote wetting and disperse reinforcement particles^22^.

**Fig. 6:**
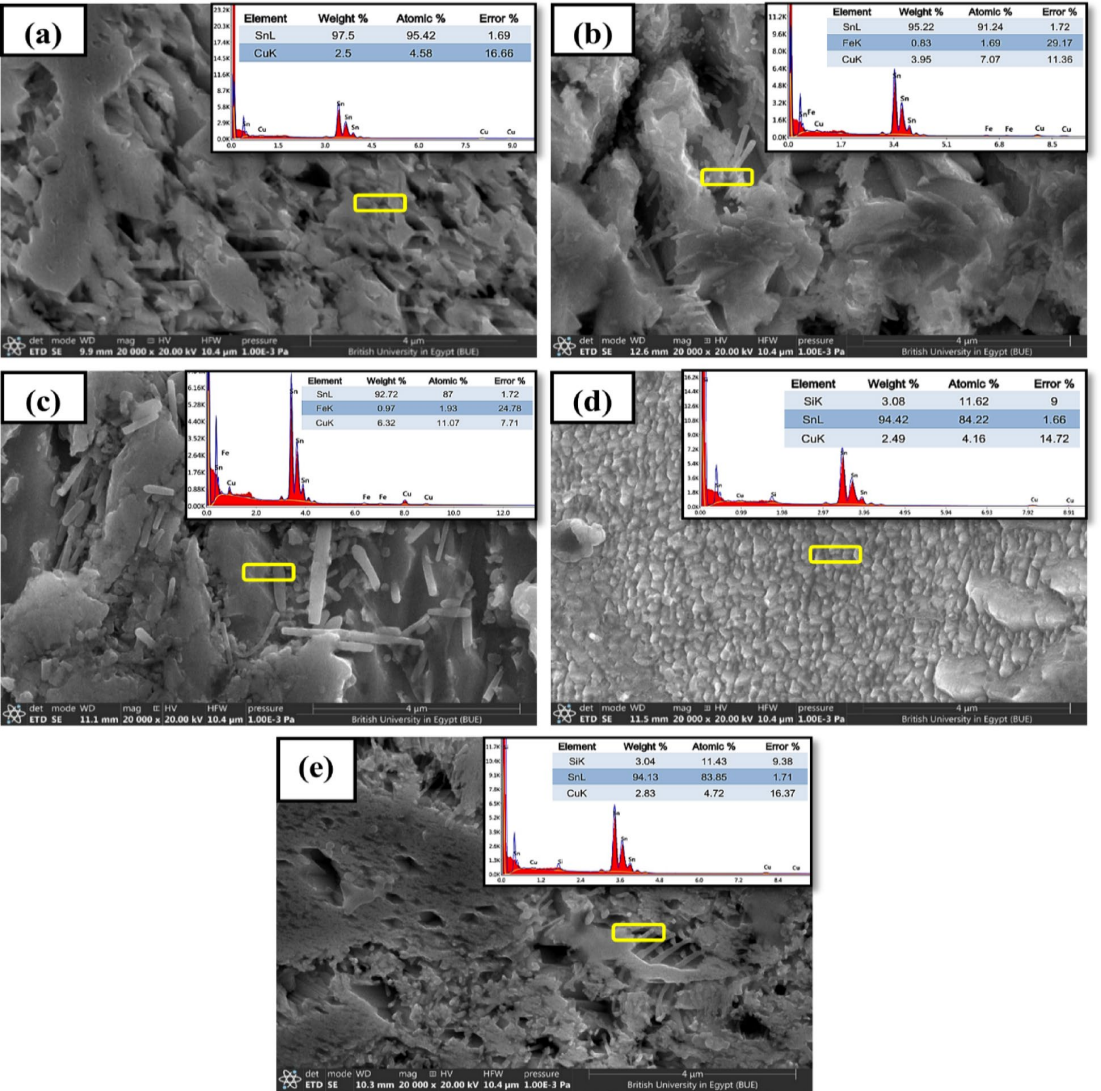
FE-SEM micrographs and the corresponding EDS analyses of (**a**) S0, (**b**) S1, (**c**) S2, (**d**) S3, and (**e**) S4 composites.

Figure 5a shows the microscopic structure of the plain solder, which has two distinct regions. The brighter region represents the β-Sn dendrites, while the darker region consists of the Sn-Cu eutectic structure of Sn and the intermetallic compound (IMC) phase, which is Cu_6_ Sn_5_. The existence of β-Sn and Cu_6_ Sn_5_ phases is confirmed through the XRD analyzes given earlier in Fig. 4 and the subsequent SEM and EDS analyses, which are depicted in Fig. 6.

Generally, the morphologies of the S_1_, S_2_, and S_3_ compounds have revealed significant refinement with the addition of WS and RHA reinforcements along with a low agglomeration of the reinforcement particles, as illustrated in Fig. 5b–d. As the concentration of RHA nanoparticles increases to 12 wt%, the agglomeration of RHA particles becomes larger with the appearance of irregular grains (Fig. 5e).

Figure 5 indicates that the WS and RHA nanoparticles may act as efficient grain refiners. This observation is confirmed through the XRD data given in Table 3, which showed that the crystallite size of the plain solder S_0_ decreased with the increase in the percent weight of WS and RHA nanoparticles.

#### SEM analysis

The interface characteristics and microstructure of the composites reflect the properties exhibited by the alloys. FE-SEM micrographs and the corresponding EDS analyses of the plain and composite solder systems are shown in Fig. 6.

The microstructural analysis illustrates how do these reinforcements influence the spreading behavior and wettability in the Sn-Cu soldering alloy. The microstructures of the S_1_, S2 and S_3_ composites show that the reinforcement particles are uniformly distributed in the matrix without notable voids and discontinuities along with good bonding between the matrix and the reinforcement particles, as well as low reinforcement agglomerations, as depicted from Fig. 6b–d. The microstructure has shown a clear interface reaction boundary in the matrix confirming good wettability of the reinforcement particles inside the matrix. The uniform dispersion of reinforcement particles throughout the matrix alloy improved both mechanical and tribological properties^28^.

It is noteworthy to note that high agglomeration of RHA particles was observed in the high percentage content of the S_4_ compound (Fig. 6e). Furthermore, it can be observed that porosities decrease with an increase in the reinforced RHA content (confirmed by Image-J processing software, version 2 (GPLv2), results shown in Appendix SI Fig. 1). These observations are in good agreement with the XRD patterns depicted in Fig. 4b and the crystallite sizes of the present compounds as tabulated in Table 3. Furthermore, SEM images, Fig. 6a–e, of the surfaces were precisely investigated to enable image-based porosity analysis. Different morphological characteristics were observed that directly relate to the degree of porosity in each sample. Sample S_0_ exhibits a relatively compact structure with minimal visible pores of 17%, indicating low porosity, supported by the high atomic percentage of SnL (95.42%) and low CuK (4.58%). Similarly, sample S_2_ shows a dense, tightly packed grain structure with uniformly distributed features and minimal voids, reflecting a low porosity of 8%^29^. On the other hand, samples S_1_ and S_3_,_4_ reveal evident voids and crack formations, implying higher porosity levels of (14, 28%, respectively) as confirmed by image-J processing software results (Appendix SI Table 1). In particular, sample S_4_ shows a fractured morphology and a scattered particle distribution, with the atomic percentage dropping to 83.85%, which is correlated with increased void content (which increased from 8% in S_2_ to 15% in S_4_ porosity) that resulted in less structural integrity due to the increased content of RHA.

Generally, all microstructures of metal-matrix composites (MMCs) have revealed significant grain refinement with the addition of Agro-waste RHA and recycled WS reinforcement particles. This is due to the good dispersion inside the matrix alloy that inhibited the devolvement of the α- grains during solidification^30^. However, agglomeration at higher reinforcement additions S_2_, S_4_ hinders uniform load transfer, reduces interfacial bonding, and traps flux residues during solidification, all of which increase porosity^31^. In Fig. 6e, the increased reinforcement contents formed micro-clusters that impeded proper solder matrix formation, consistent with observed microstructural irregularities.

Solidification behavior has resulted in a grain refinement and nonhomogeneous nucleation slag particles at the center^32^. A clear and discernable dendritic microstructural propagation reflects a unique and rapid solidification-induced grain refinement mechanism^33^. It is also expected to show unique behavior in many soldering applications as a result of the improved wettability and gained thermal stability characteristics.

### Mechanical behavior analysis

The effect of changes in the percentage of WS and RHA added to Sn–0.7Cu solder can be analyzed by determining the mechanical behavior of these composite samples subjected to different types of tests. In this study, composite samples with different WS and RHA are analyzed using two important mechanical properties, namely tensile testing and microhardness measurements.

#### Tensile strength testing

**Fig. 7:**
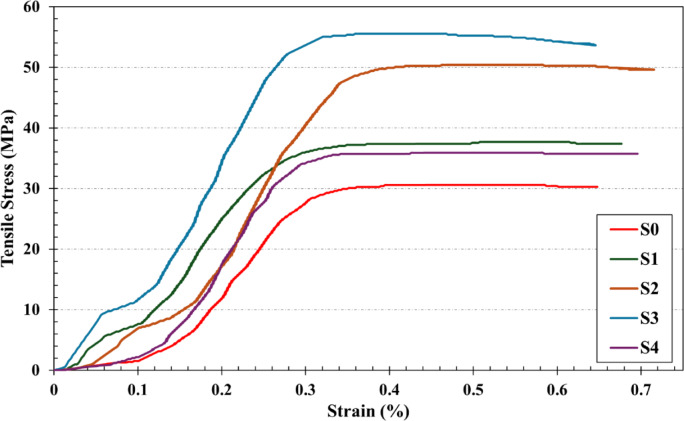
Tensile stress–strain curves of S0, S1, S2, S3, and S4 composites.

Testing was performed on five different composite samples S_0_, S_1_, S_2_, S_3_, and S4 to determine their mechanical strength parameters. Figure 7 illustrates the tensile stress–strain curves for all composite samples at a constant strain rate of $\:1.7\times\:{10}^{-4}{\:\text{s}}^{-1}$. In general, these curves are significantly dependent on the content of WS and RHA. It is also significant to compare the effects of the use of Agro-waste RHA and WS with conventional reinforcements reflecting sustainability concerns.

The tensile strengths of the composites continuously increase as the fraction of WS and RHA reinforcements increases by weight, demonstrating that WS and RHA reinforce the present Sn–0.7Cu solder. The results of this figure reveal that the inclusion of WS and RHA nanoparticles improved stress adaptation.

The ultimate strength also increased, pointing to improved mechanical qualities. These results are consistent with the existing literature from various studies^34–37^. The strengthening of composites occurs through two mechanisms; the first mechanism involves increased resistance to plastic deformation of the matrix material under load, attributed to direct load transfer to reinforcement particles at the interface limits of the reinforcements^38^.

The second mechanism involves the effective binding of reinforcement particles to the matrix, attributed to the high wettability of the WS and RHA particles^6^. The strength of WS and RHA metal matrix composites is improved by these two mechanisms.

Table 4 summarizes the tensile parameters of the S_0_, S_1_, S_2_, S3 and S_4_ composites, involving ultimate tensile strength (σ_u_), ductility (ε_t_), and modulus of toughness (U__T_). The tabulated data show that increasing the concentration of WS and RHA concentration leads to higher σ_u_ and ε_t_ values in composites compared to the base matrix material.

**Table 4:** Tensile properties: σ_u_, ε_t_, and U__T_ of Sn-0.7Cu-x (WS, RHA) compounds.

Alloy	σ_u_ (MPa)	ε_t_ (%)	U__T_ (MJ/m^3^)
**S**_**0**_	30.6 ± 2%	0.648 ± 2%	13.23
**S**_**1**_	37.7 ± 3%	0.677 ± 2%	19.14
**S**_**2**_	50.4 ± 3%	0.715 ± 2%	24.74
**S**_**3**_	55.6 ± 3%	0.646 ± 2%	26.40
**S**_**4**_	35.9 ± 3%	0.696 ± 2%	17.75

The toughness values of Sn–0.7Cu composite solders, Table 4, exhibit significant variation with the reinforcement additions, indicating a strong influence of the type and content on energy absorption capacity. The base alloy sample (S_0_) demonstrated the lowest toughness of 13.23 MJ/m^2^, which increased considerably with the addition of 6 wt% WS to 19.14 MJ/m^3^, which constitutes a 44.7% improvement. A further increase to 12 wt% WS added 24.74 MJ/m^2^ toughness, which constitutes an improvement of 87%, suggesting that WS particles enhance crack resistance and energy absorption during deformation. This improvement can be attributed to effective load transfer between the matrix and WS particles, along with observed grain refinement and dispersion strengthening mechanisms introduced by such reinforcements as presented in Fig. 6a–c.

However, the use of RHA yielded a different trend. While the addition of 6 nominal wt% RHA reinforcement led to a notable improvement in toughness value, 26.4 MJ/m^2^, which constitutes 99.5% improvement. Another increase, 12 nominal wt% RHA reinforcement, resulted in a decline to 17.75 MJ/m^2^.

This suggests that there is an optimal content existing, beyond which agglomeration and inadequate bonding, Fig. 6d,e, between reinforcement particles and the solder matrix can induce stress concentration and premature failure. These findings underscore the importance of optimizing the type and quantity to achieve optimal mechanical performance in sustainable Sn–Cu composite solders.

**Fig. 8:**
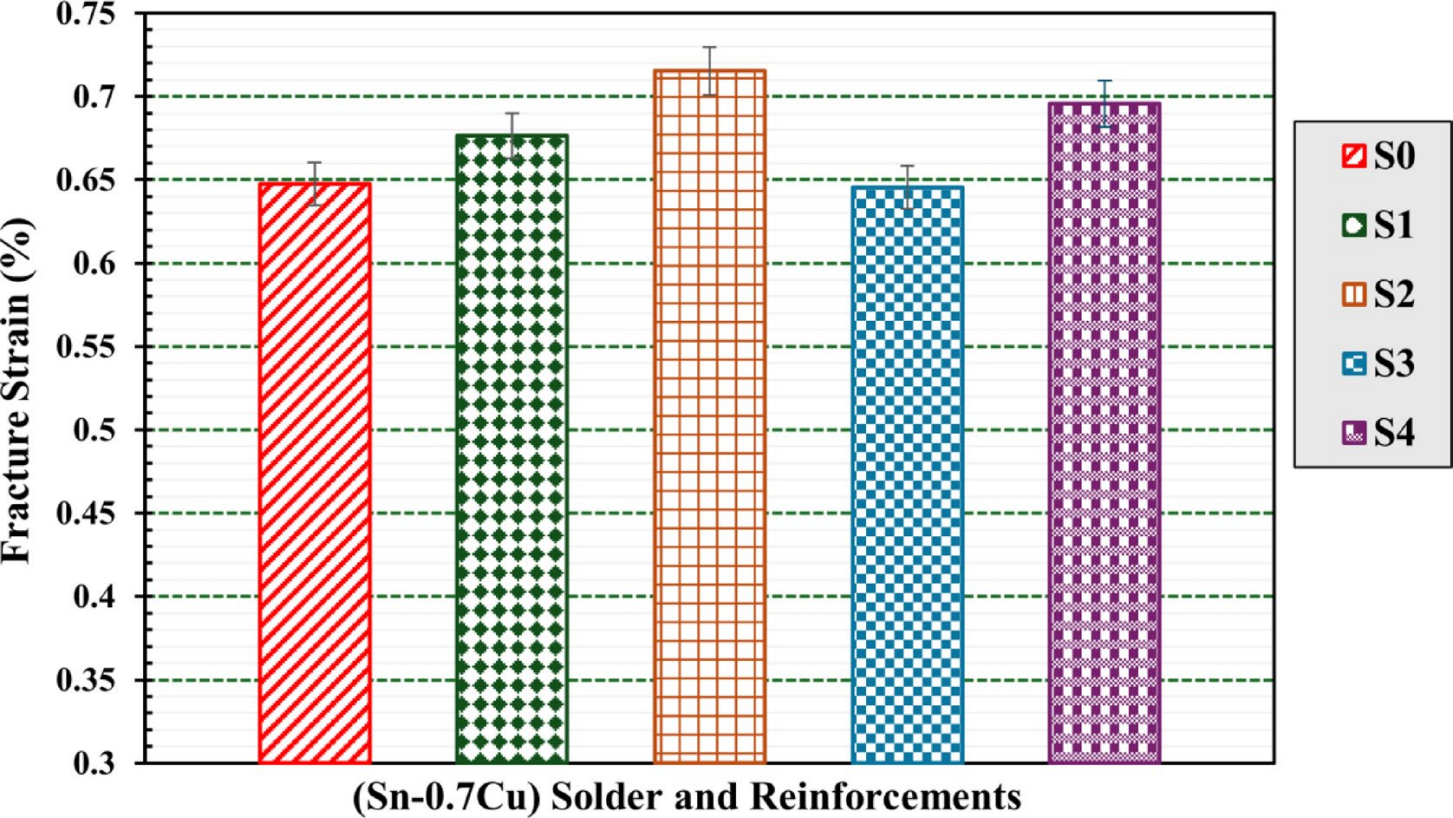
Bar graph of fracture strain of S0, S1, S2, S3, and S4 composites.

Figure 8 depicts ultimate strength (σ_u_) determined from the tensile stress−strain curves of the S_0_, S_1_, S_2_, S3 and S_4_ composites. It is observed that the ultimate strength is lower in pure S_0_ samples compared to other percentages of WS and RHA content. The ultimate strength of the Sn-0.7Cu-xWS presented a kind of gradually increasing trend with increasing WS content. It increased significantly increased from 30.6 ± 2% MPa for S_0_ to 37.3 ± 3% MPa and 50.4 ± 3% MPa for S_1_ and S_2_, respectively. This discovery further emphasizes the potential for the strength of the composite material.

This improvement in ultimate strength was due to the effective dispersion of WS nanoparticles. As a result, such Sn–0.7Cu alloy matrix reinforced with WS nanoparticles can well resist high tensile stresses and achieve higher ultimate tensile strength, as shown in Fig. 8.

However, the ultimate strength of the Sn-0.7Cu-xRHA showed an initial increase followed by a subsequent decrease. It increased significantly increased from 30.6 ± 2% MPa for S_0_ to 55.6 ± 3% MPa for S_3_, while it decreased again to 35.9 ±3% for S_4_. This shows that a large content of RHA nanoparticles is not suitable to improve the tensile strength of the present Sn–0.7Cu solder. The observed improvement in the tensile strength of the present Sn–0.7Cu solder is attributed to the fact that the filler recycled WS and RHA possess higher strength by offering more resistance. The WS and RHA nanoparticles act as barriers to the movement of dislocations when load is applied. Similar observations were found in other studies for the dispersions of fly ash particles^39–41^.

**Fig. 9:**
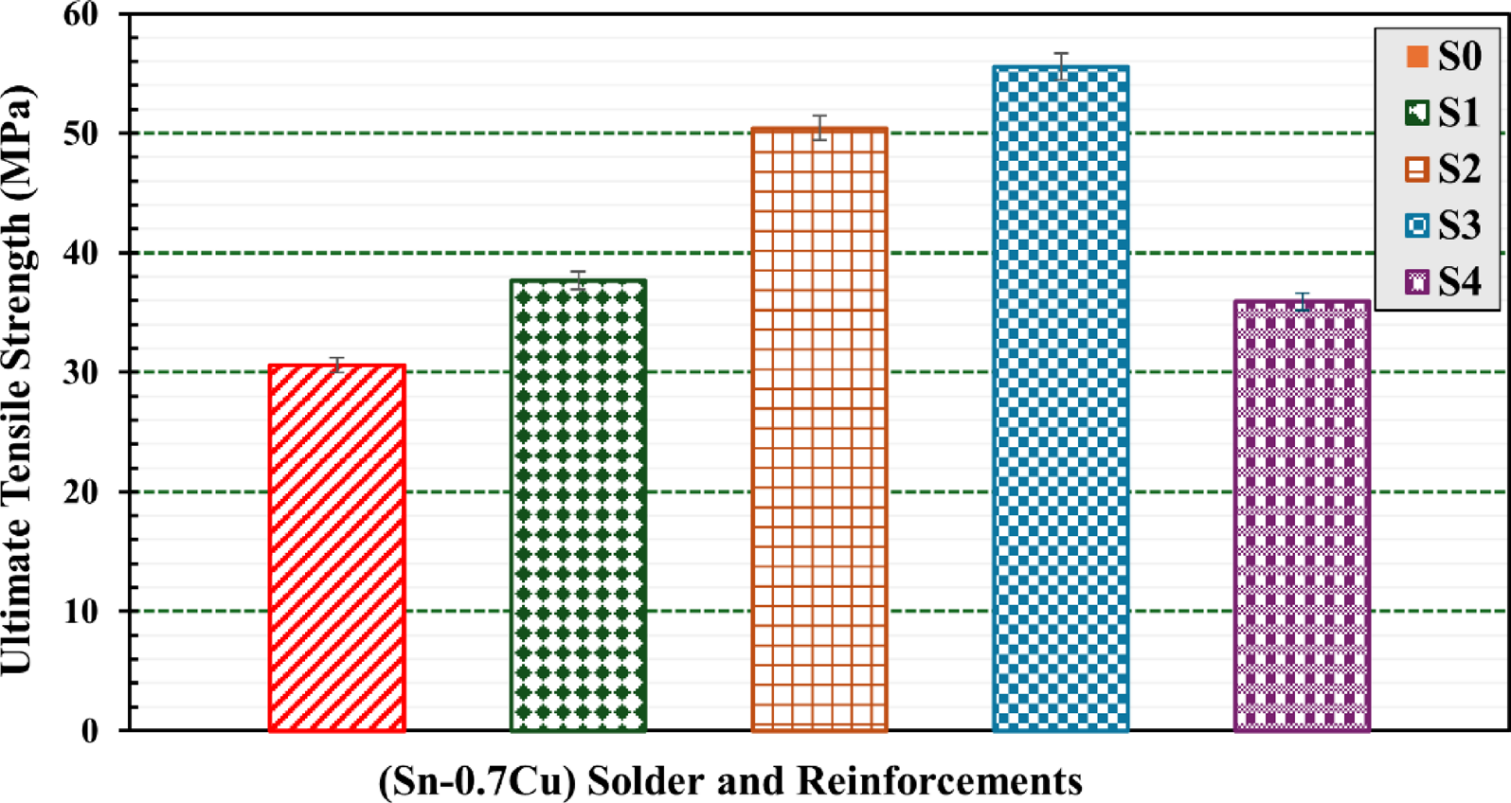
Bar graph of ultimate tensile strength of S0, S1, S2, S3, and S4 composites.

However, the main reason for the decrease in the tensile strength of the S_4_ compound containing high nominal content (12 wt% RHA) is the poor wettability of the RHA particles with the matrix due to the reduction in the interfacial bonding between the RHA particles and the matrix. Therefore, a high agglomeration of RHA particles and excessive porosities and voids within the matrix were observed, as shown earlier in Fig. 6e. This explanation is confirmed by the data given in Table 3. A similar tendency was found in the Al hybrid composite by SiC and fly compositions^42^. This is also evident in other studies as well^6,43^ in which the tensile strengths of the compounds increase as their particle reinforcement sizes increase but decrease beyond the optimum level.

However, a vital issue to consider is the ductility of materials. Increased ductility could ease the material’s ability to bend plastically under high stress. The effect of weight content of WS and RHA on percentage elongation of the present Sn-0.7Cu solder is illustrated in Fig. 9. The present composites, especially with larger weight percentages of WS and RHA, are observed to exhibit ductile fracture behavior after a certain level of elongation.

This implies that while the increase in the dispersion content of WS and RHA increases the tensile strength of the Sn-0.7Cu solder, they also increase its ductility. This could be attributed to the fact that the WS and RHA particles are thermodynamically stable in the matrix and reduce the embrittlement effects^44^ of the particles, thus improving the ductility of the Sn–0.7Cu solder. These results are in good agreement with other studies^21,45^. On the other hand, other studies found dissimilar observations with our results for various weight% of fly ash reinforcement^46–48^.

#### Hardness analysis

**Fig. 10:**
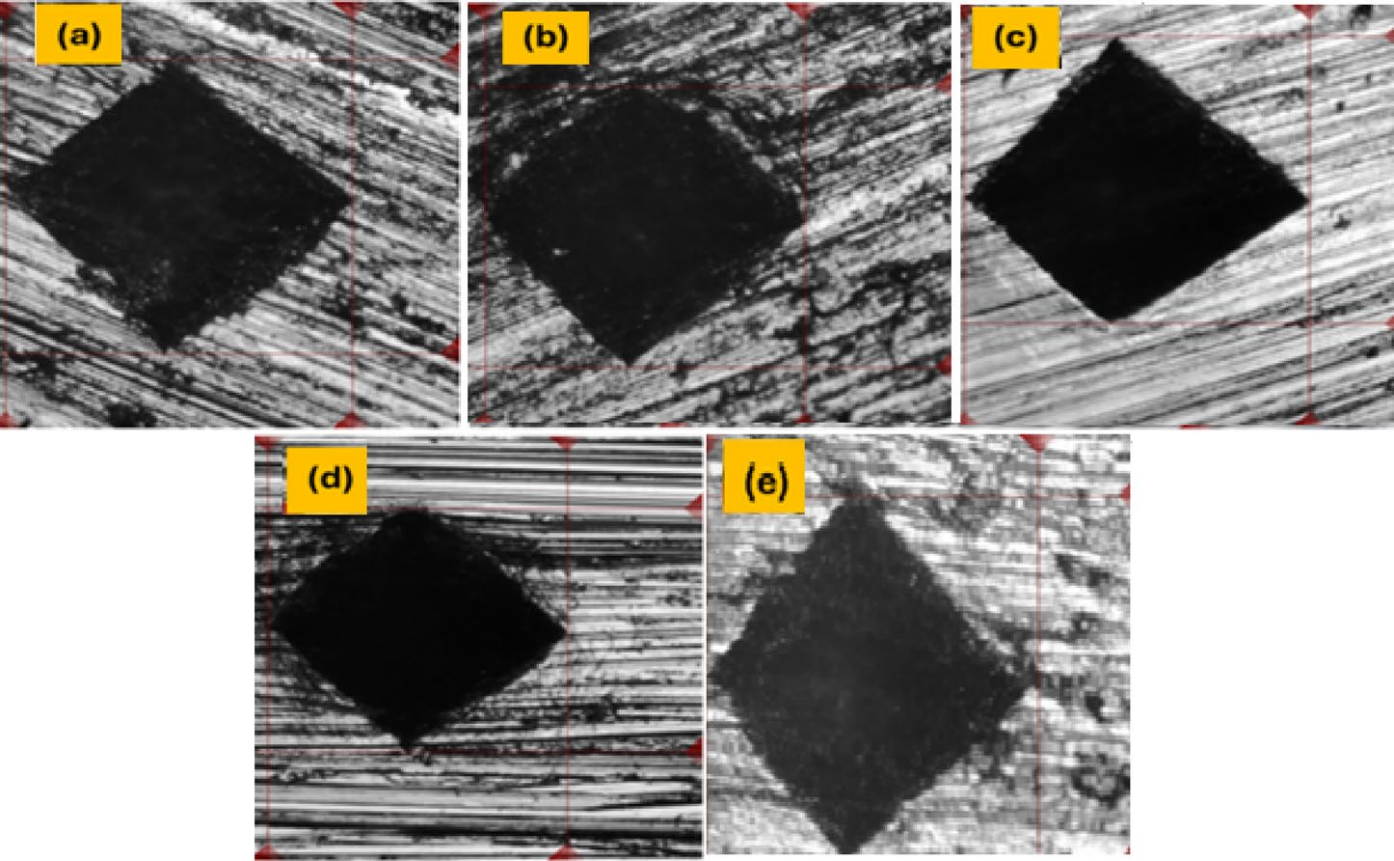
Optical images of Vickers microhardness indentation trace of: (**a**) S0, (**b**) S1, (**c**) S2, (**d**) S3, and (**e**) S4 composites.

Hardness testing is recognized as an effective technique for measuring the mechanical characteristics of materials and alloys. The microstructure of materials is widely recognized to significantly affect their hardness^49^. Figure 10 shows optical images of Vickers microhardness indentation trace of the S_0_, S_1_, S_2_, S3 and S_4_ composites.

**Fig. 11:**
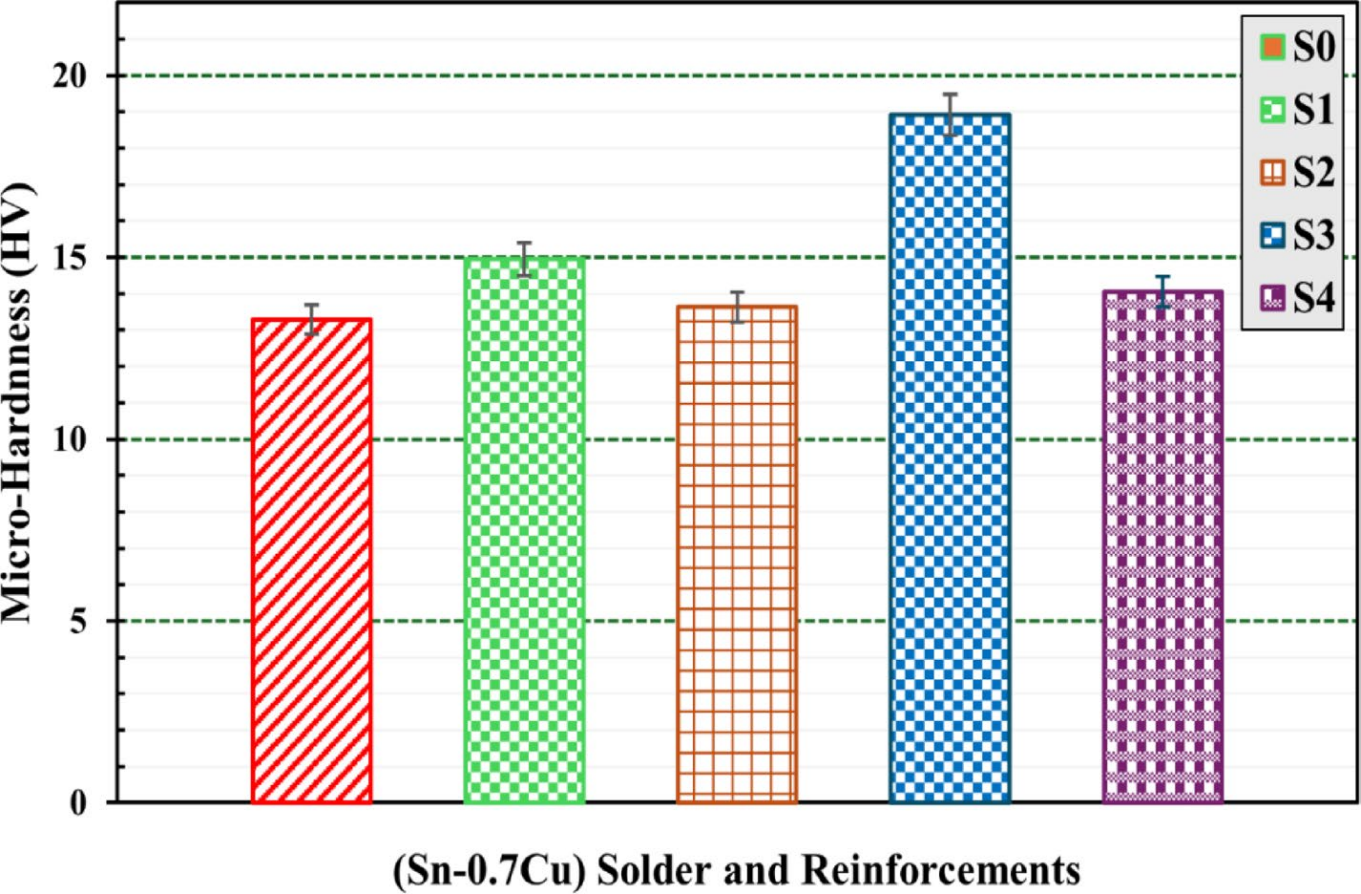
Bar graph of microhardness of S0, S1, S2, S3, and S4 composites.

The hardness values are presented as an average of five taken at different locations in the samples. Vickers microhardness values at room temperature for different composites investigated are shown in Fig. 11 and summarized in Table 5. Figure 11 clearly indicates that the Vickers microhardness values of the S_1_, S_2_, S3 and S_4_ composites exceed those of the plain solder S_0_.

The hardness of plain solder S_0_ increased significantly after 6% of WS and RHA were added. In particular, there was an obvious difference in hardness between plain solder S_0_, which measured 13.29 ± 0.54 Hv, and a composite that contained 6% WS (S_1_), which measured 14.94 ± 0.76 Hv.

The Vickers hardness value of the composite containing 6% RHA (S_3_) has the maximum value (18.92 ± 0.66 Hv) among the other concentrations, since its increase represents ≈ 42.5% larger than that of the plain solder S_0_. This occurs due to increases in the surface area of the matrix, and thus the grain sizes are reduced. The presence of such a hard surface offers more resistance to plastic deformation, leading to an increase in hardness^50^.

**Table 5:** Microhardness results of Sn-0.7 Cu-x (WS, RHA) composite solders.

Alloy	HV (kg/mm^2^)	SD
**S**_**0**_	13.29	0.54
**S**_**1**_	14.94	0.76
**S**_**2**_	13.63	0.47
**S**_**3**_	18.92	0.66
**S**_**4**_	14.05	0.52

The higher microhardness values of the composites can be attributed to reinforcements impeding dislocation motion^51^. Furthermore, this can be explained by the fact that the reinforcement nanoparticles of WS and RHA were stiffer than the matrix, which prevented plastic deformation. It was the main reason for WS and RHA particles’ presence due to their lightweight and high stability against indentation load. Similar findings were made by Radhakrishna et al. for SiC particle reinforcements^52^.

Furthermore, the addition of 12% nominal weights of WS and RHA resulted in slightly higher hardness compared to the plain solder S_0_. In particular, there are small differences in hardness between plain solder S_0_, which measured 13.29 ± 0.54 Hv, and compounds that contained 12 wt% of WS (S_2_), and RHA(S_4_), which measured 13.63 ± 0.47 Hv and 14.05 ± 0.52 Hv, respectively. These results are in good agreement with other studies^53,54^.

The reason for the increased hardness of the S_1_ and S_3_ composites can be attributed to the amazing bonding of WS and RHA particles within the base solder matrix. Although they had lower WS or RHA contents compared to the other samples (S_2_ and S_4_), S_1_ and S_3_ showed higher hardness, suggesting that lower WS or RHA contents helped greatly in boosting the grain refinement mechanism. This highlights an optimal ratio of reinforcements in the base solder matrix. Furthermore, dislocation hardening can also be considered a reason for the increased hardness of this metal matrix composite (MMC)^55^. The applied load was transferred directly to the reinforcement particles. Rather than just sustaining the load, these reinforcement particles have restricted the geometric deformation of the plastic.

This created enough dislocations within the matrix to handle the applied load, thus increasing the dislocation density, which resulted in hardening of the dislocation. Dispersion hardening and Orowan strengthening can also be attributed as reasons for the increased hardness of this MMC from its baseline solder. Although an increase in plastic deformation was expected as the percentage of reinforcement increased, the hardness obtained for samples with 12 wt% WS (S_2_) and 12 wt% RHA (S_4_) was comparatively lower. This is due to the minor agglomeration of reinforced particles in the Sn–0.7%Cu matrix caused by irregular mixing^55^.

These results underscore the importance of WS and RHA nanoparticles in improving the mechanical characteristics of Sn–Cu solders, making them ideal for several demanding applications^56^.

The summarized data in Table 6 clearly illustrate the significant influence of the incorporation of recycled welding slag and rice husk ash (RHA) in the microstructural, thermal, and mechanical behavior of Sn–Cu solder composites. In particular, the progressive refinement of crystallite size, especially in samples S_1_–S_3_, is strongly with increased dislocation density, indicating enhanced defect-induced strengthening mechanisms.

**Table 6:** A summary table comparing all sample properties.

Alloy	Crystallite diameter (nm)	Dislocation density (nm^− 2^)	Melting Tenp. (°C)	Pasty range (°C)	Ultimate tensile strength (MPa)	_Strain_ (%)	Micro-Hardness (kg/mm^2^)
**S**_**0**_	298.50	1.12 × 10^− 5^	226.10	22.12	30.6±2%	0.648±2%	13.29
**S**_**1**_	39.15	6.52 × 10^− 4^	224.65	24.14	37.7±3%	0.677±2%	14.94
**S**_**2**_	39.11	6.54 × 10^− 4^	225.44	23.47	50.4±3%	0.715±2%	13.63
**S**_**3**_	47.32	4.47 × 10^− 4^	227.54	21.73	55.6±3%	0.646±2%	18.92
**S**_**4**_	194.62	2.64 × 10^− 5^	227.68	27.04	35.9±3%	0.696±2%	14.05

This microstructural evolution is further reflected in the improved microstructure and ultimate tensile strength and microhardness values, with S_3_ showing optimal performance due to its balanced structural integrity and grain boundary strengthening. Additionally, the slight elevation in the melting temperature and narrow pasty range of these modified solders suggest improved thermal stability and a more controlled solidification profile, which is a desirable trait for practical soldering applications.

Although S_4_ showed an anomalous increase in crystallite size, likely due to particle agglomeration at higher additive ratios, its mechanical properties remain competitive. In general, the findings validate the potential of eco-friendly reinforcements, such as RHA and recycled WS, to enhance solder properties, promoting sustainability without compromising performance^57–59^.

## Conclusions

This study examined the impacts of dispersing WS and RHA nanoparticles on the microstructural and mechanical characteristics of the Sn–0.7Cu solder alloy, with the main conclusions outlined as follows:

- The inclusion of WS and RHA nanoparticles significantly improved the mechanical characteristics of the present Sn–0.7Cu solder, involving the ultimate tensile strength, ductility, and microhardness.
- The inclusion of RHA nanoparticles exhibited mechanical characteristics of the present Sn–0.7Cu solder than those of WS nanoparticles.
- The mechanical characteristics of the Sn–0.7Cu solder increased as the weight fraction of the WS and RHA nanoparticles increased to a certain limit. However, for composites with more than 6 nominal wt% of recycled RHA nanoparticles, the mechanical characteristics were slightly affected or sometimes decreased.
- The reinforced Sn–0.7Cu-6RHA composites exhibited superior tensile strength and hardness values compared to the unreinforced Sn–0.7Cu solder.
- The SEM images demonstrate that the recycled WS and RHA nanoparticles are rather evenly dispersed throughout the composite material at lower dispersion contents.
- The good dispersibility of the WS and RHA nanoparticles within the Sn matrix improved the hardness and tensile behavior of the Sn–0.7Cu compound.
- The grain refining of the resulted microstructure of the reinforced Sn–0.7Cu solder is the primary strengthening mechanism responsible for the improvement of mechanical and thermal characteristics.

Incorporation of WS and RHA, derived from industrial and agricultural waste, into Sn–0.7Cu solder alloys improved chosen mechanical and thermal properties at optimized nominal weight fractions. These findings imply potential concerns regarding sustainable, resource-efficient solder composites. However, applying on large-scale requires further validation, regarding joint reliability, process control, and compatibility with existing soldering systems. Future studies should also include standardizing preprocessing methods and comprehensive environmental and economic assessments to better evaluate overall sustainability and industrial feasibility of such waste-reinforced solder materials.

## Supplementary Information

Below is the link to the electronic supplementary material.

Supplementary Material 1

## Data Availability

The datasets used/analyzed during the current study are available from the corresponding author upon reasonable request.
