# Supplementary material for: Enhancing sustainability in soldering: effects of recycled welding slag and rice husk ash on microstructural, thermal, and mechanical properties of Sn–Cu composite solders
Source: Sci Rep. 2025 Nov 28;15:42911. doi: 10.1038/s41598-025-27369-z (PMC12672664; doi:10.1038/s41598-025-27369-z)
Supplement: Supplementary file 1 — Supplementary Material 1 [file 41598_2025_27369_MOESM1_ESM.pdf]

Appendix I

[A]

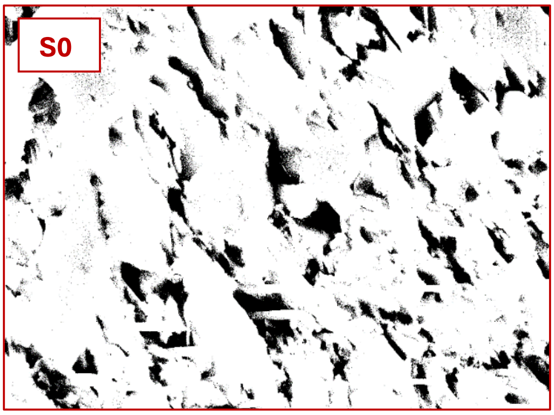

[B]

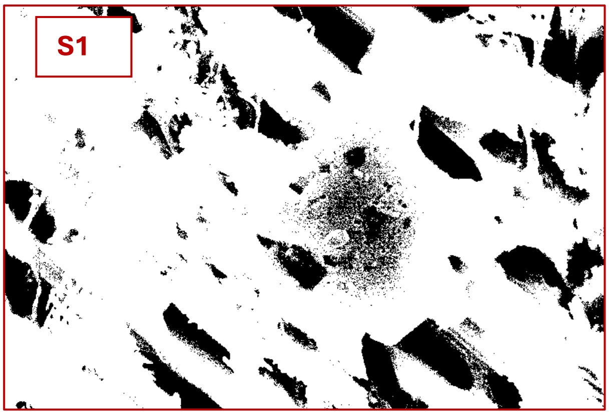

[C]

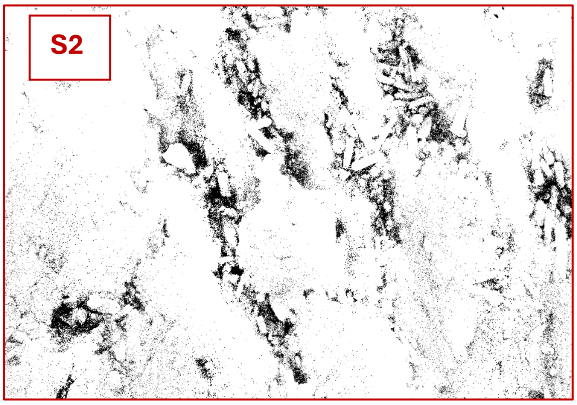

[D]

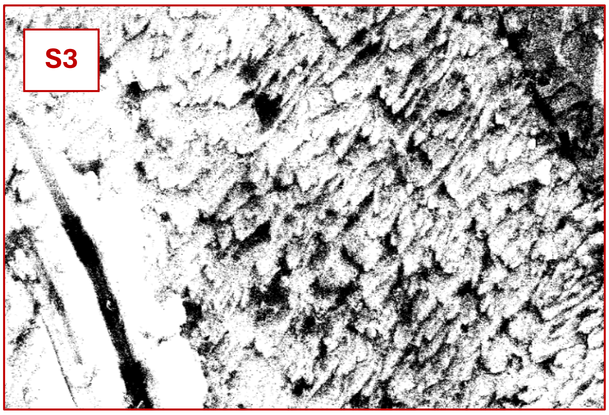

[E]

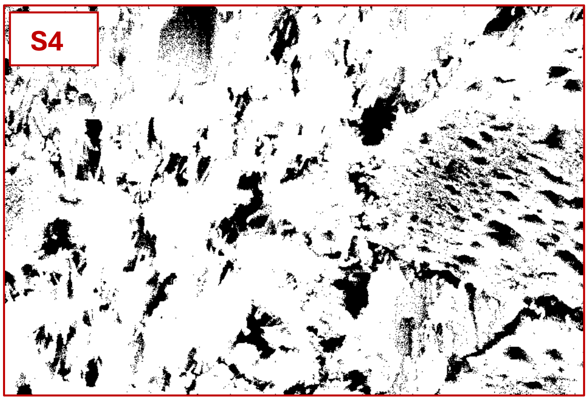

SI Fig.1 Image-J software results for all test samples from A to E (S0:S4)

SI Table 1 Image-J software porosity results

Sample	Porosity, %
S_0	17
S_1	14
S_2	8
S_3	27
S_4	15
